# Long-Term Follow-Up Evaluation of Renal Function in Patients with Chronic Kidney Disease Undergoing Cardiac Surgery

**DOI:** 10.1155/2016/9680718

**Published:** 2016-06-30

**Authors:** Eduesley Santana-Santos, Felipe Kenji Oshiro Kamei, Tarcísia Karoline do Nascimento, Anas Abou Ismail, Jurema da Silva Herbas Palomo, Marcia Cristina da Silva Magro, Fátima Gil Ferreira, Larissa Bertacchini de Oliveira, Adriano Rogério Baldacin Rodrigues, José Jayme Galvão de Lima

**Affiliations:** ^1^Nursing Department, Heart Institute (InCor), Hospital das Clínicas da Faculdade de Medicina da Universidade de São Paulo, São Paulo, SP, Brazil; ^2^Vanderbilt University, Nashville, TN, USA; ^3^Universidade de Brasília, Brasília, DF, Brazil; ^4^Heart Institute (InCor), Hospital das Clínicas da Faculdade de Medicina da Universidade de São Paulo, São Paulo, SP, Brazil

## Abstract

*Background*. Acute kidney injury (AKI) is a common complication of cardiac surgery but its long-term consequences, in patients with chronic kidney disease (CKD), are not known.* Methods*. We compared the long-term prognoses of CKD patients who developed (*n* = 23) and did not develop (*n* = 35) AKI during the period of hospitalization after undergoing coronary artery bypass graft (CABG). Fifty-eight patients who survived (69.6 ± 8.4 years old, 72% males, 83% Whites, 52% diabetics, baseline GFR: 46 ± 16 mL/min) were followed up for 47.8 ± 16.4 months and treated for secondary prevention of events.* Results*. There were 6 deaths, 4 in the AKI+ and 2 in the AKI− group (Log-rank = 0.218), two attributed to CV causes. At the end of the study, renal function was similar in the two groups. One AKI− patient was started on dialysis. Only 4 patients had an increase in serum creatinine ≥ 0.5 mg/dL during follow-up.* Conclusion*. CKD patients developing AKI that survived the early perioperative period of coronary intervention present good renal and nonrenal long-term prognosis, compared to patients who did not develop AKI.

## 1. Introduction

Acute kidney injury (AKI) is a serious complication of a variety of conditions and is associated with increased mortality and longer periods of hospitalization [1, 2]. It is well known that preexisting chronic kidney disease (CKD) is an important risk factor for the development of postoperative AKI [3]. However, much less is known about the long-term renal and nonrenal consequences of postoperative AKI in patients with CKD undergoing major surgical interventions [4].

In a previous double-blind prospective work [5], we showed that high IV doses of N-acetylcysteine (NAC) reduced the incidence of AKI in high-risk patients with CKD undergoing coronary intervention. In the present investigation, we report on the long-term (up to six years) renal function and prognosis of patients of that study who survived the early postoperative period.

## 2. Methods

The study was conducted in accordance with the Helsinki postulates and was approved by the Institutional Ethics Board (number 3303/013/098). All the patients provided informed signed consent. This was an observational analysis of data collected in 58 CKD patients who survived the early postoperative period of elective coronary artery bypass graft (CABG) intervention. Patients were part of a larger population of 70 individuals with CKD, stage 3 or 4, were randomized to receive an IV dose of either NAC 200 mg/kg or a placebo during operation, and were followed up until death or discharge from the hospital [5]. Six patients died during the immediate postoperative period and 6 were lost to follow-up, leaving 58 individuals that were finally included in this study. Patients were followed up from the time of hospital discharge until death. End point was either the initiation of dialysis or death by any cause.

Patients were seen at least once a year at the hospital clinic and received a standard treatment consisting of renin-angiotensin inhibitors, aspirin, beta-blockers, and statins as recommended for secondary prevention of cardiovascular events. Insulin, hypoglycemic drugs, diuretics, and other antihypertensive drugs were also administered at the discretion of the attending doctors. Control of body weight and smoking cessation were encouraged. The causes of death were analyzed by review of the charts. AKI was defined by the Kidney Disease: Improving Global Outcomes (KDIGO) [6] criteria stage 1, 2, or 3. Glomerular filtration rate (GFR) was estimated by the MDRD method [7].

For analysis of the results, we used SPSS statistical package (version 20.0; IBM, Armonk, USA). Results are expressed as means ± standard deviation and percentages. All analyses were two-tailed. Student's *t*-test and chi-square test were used as indicated. Survival curves were constructed by the Kaplan-Meier method and compared by the Log-rank method. Cox proportional model was used to assess factors that independently influenced death and progression to dialysis.

## 3. Results

Among the 58 patients discharged after the operation, 23 (40%) had developed postoperative AKI while 35 (60%) did not.  Table 1 shows the main characteristics of the total population as well as of patients with and without postoperative AKI.

Most patients were above 60 years old. The mean follow-up was 47.8 ± 16.4 months (range: 23 to 72). There was predominance of Caucasian males. Smoking, diabetes, and, particularly, other cardiovascular (CV) diseases were prevalent. In patients who developed AKI, serum creatinine was higher and GFR lower at the time of discharge from the hospital. On the other hand, at the final evaluation, creatinine and GFR were comparable between groups. In all other aspects, the groups were well balanced. During follow-up, 6 patients died, 4 in the AKI+ and 2 in the AKI− group, while 1 subject that did not develop AKI was admitted to a dialysis program. These differences were not significant. The causes of deaths were the following: AKI+: cancer (2 cases), myocardial infarction (1 case), and infection (1 case); AKI−: myocardial infarction (1 case) and chronic obstructive pulmonary disease (1 case).

**Figure 1:**
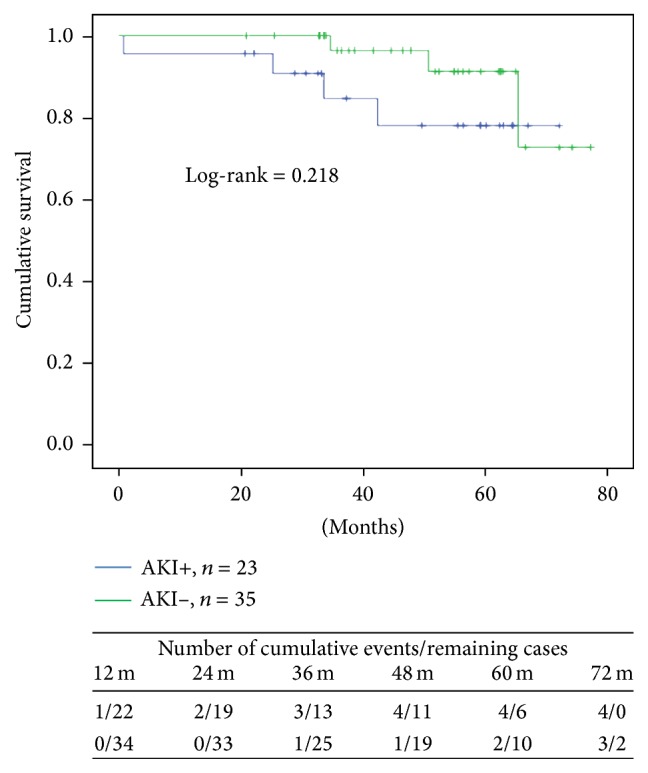
Long-term survival curves of patients with and without AKI.

Figure 1 shows the long-term survival curves of patients with and without postoperative AKI. There was no difference between groups (Log-rank = 0.218). The Cox proportional model that included age, diabetes, other CV diseases, AKI, and use of perioperative NAC failed to identify independent risk factors associated with either death or death plus initiation of dialysis treatment.

Table 2 compares the values of variables collected at the time of discharge from the hospital and at the final period of observation in patients who did and did not develop postoperative AKI. Systolic and diastolic blood pressure and hemoglobin concentration increased at the last observation period in both groups. Only in four subjects was an increase in serum creatinine ≥ 0.5 mg/dL observed: 1 in a patient with AKI and 3 in patients that did not develop AKI (including 1 that was started on dialysis). GFR increased with time in patients with postoperative AKI and fell in patients that did not develop that complication.

## 4. Discussion

The main finding of this study was that the long-term survival of patients with CKD undergoing a major cardiac operation that survived the early postoperative period was not influenced by postoperative AKI. In addition, AKI did not influence the renal prognosis since renal function increased significantly at the end of the period of observation. Moreover, no patient in that group needed dialysis treatment and only one subject experienced an increase in serum creatinine at the conclusion of the study of at least 0.5 mg/dL. These results differ from those reported in a meta-analysis by Coca et al. [8]. In 15 out of 48 studies in the literature reporting long-term data, the authors observed that after an episode of AKI the incidence rate of mortality was 8.9 deaths/100 person-years and was 4.3 deaths/100 person-years in individuals that did not present AKI. Although the incidence of terminal renal failure appears to be elevated, the relative risk to progress to that condition after AKI could not be determined because of the lack of appropriate controls in all studies reviewed. A strong point in our report is that here we included a control group of patients that underwent the same procedure, under similar conditions, and that did not develop AKI after the operation.

In a large unicentric work that included 1826 individuals undergoing coronary intervention, McCullough et al. [9] observed that incidence of acute renal failure was 144.6/1000 while the incidence of severe renal failure in need of dialysis was much lower, less than 1%. However, the long-term consequences of acute renal failure were not reported.

In another study, in patients critically ill admitted to the ICU, the incidence of severe acute renal failure was 11/100.000 population-years [10]. Nearly 80% of patients recovered renal function on follow-up, including those that needed dialysis. On the other hand, the incidence of AKI in patients undergoing coronary percutaneous intervention is low [11]. However, in the same study, acute renal failure was strongly correlated with death after discharge from the hospital. Finally, a large retrospective study, comprising more than 140,000 patients with myocardial infarction, showed that AKI had an independent and graded association with long-term mortality [12].

The more favorable results reported here may be related to the milder degree of renal dysfunction in our patients (only one patient had AKI stage 3) and to the small size of our sample. The possible effect of the perioperative NAC administration was not statistically significant. We may also speculate that the universal use of cardioprotective drugs and the adequate control of blood pressure could also have influenced the results. In spite of all this, considering that this was a high-risk population and that all subjects had severe coronary artery disease, the results may be relevant.

We acknowledge that this study has limitations. The number of patients and the incidence of events were small and the number of individuals lost to follow-up was high, which reduced the statistical power of our sample. On the other hand, this investigation included a control group that is lacking in the majority of the reports on the same subject.

In conclusion, we showed that CKD patients developing AKI, who survived the early perioperative period of coronary intervention, present good renal and nonrenal long-term prognosis, compared to patients who did not develop AKI. Only two CV deaths and just one occurrence of advanced renal disease in need of dialysis happened during a mean follow-up of 48 months. However, it should be stressed that the results presented here could have been influenced by the small number of patients studied. Larger prospective investigations are needed to clarify this important area.

## Figures and Tables

**Table 1:** Long-term characteristics of CKD patients who had undergone CABG who developed and did not develop postoperative AKI.

Variable	Total, *n* = 58	AKI+, *n* = 23	AKI−, *n* = 35	*p* value
Age (years)	69.6 ± 8.4	70.9 ± 7.5	68.8 ± 8.9	0.84
Sex (males)	42 (72%)	17 (74%)	25 (71%)	0.84
Caucasian	48 (83%)	19 (83%)	29 (83%)	0.98
Smoking	22 (38%)	9 (39%)	13 (37%)	0.88
Diabetes	30 (52%)	15 (62%)	15 (43%)	0.09
Other CVD	53 (91%)	21 (91%)	32 (91%)	0.98
Follow-up months	47.8 ± 16.4	45.2 ± 18.9	49.6 ± 14.6	0.31
Death	6 (15%)	4 (17%)	2 (5.7%)	0.15
End-stage CKD	1 (3.4%)	0 (0%)	1 (2.9%)	0.41
SBP discharge	121 ± 14	124 ± 15	120 ± 13	0.24
SBP final	135 ± 21	137 ± 20	134 ± 22	0.53
DBP discharge	73 ± 11	71 ± 9	74 ± 12	0.38
DBP final	79 ± 9	79 ± 11	79 ± 10	0.99
BMI discharge	27.3 ± 4.3	28.1 ± 4.8	26.8 ± 3.9	0.27
BMI final	27.4 ± 5.2	27.4 ± 5.7	27.4 ± 5.0	0.99
Creatinine discharge	1.7 ± 0.6	2.1 ± 0.6	1.4 ± 0.4	0.0001
Creatinine final	1.6 ± 0.9	1.7 ± 1.1	1.6 ± 0.7	0.69
GFR discharge	46 ± 16	37 ± 14	52 ± 15	0.0001
GFR final	47 ± 13	46 ± 13	48 ± 13	0.70
Hb discharge	9.2 ± 1.7	8.8 ± 1.2	9.5 ± 1.9	0.16
Hb final	13.5 ± 1.9	13.4 ± 2.7	13.4 ± 1.8	0.99

Data are expressed as mean ± standard deviation and absolute and relative frequencies. AKI: acute kidney injury; CVD: cardiovascular disease; CKD: chronic kidney disease; SBP: systolic blood pressure; DBP: diastolic blood pressure; BMI: body mass index; GFR: glomerular filtration rate; Hb: hemoglobin.

**Table 2:** Characteristics of CKD patients who had undergone CABG who developed and did not develop AKI during the postoperative period at discharge and final evaluation.

Variable	Discharge, *n* = 58	Final, *n* = 52	*p* value
Death AKI+	0	4	0.80
Death AKI−	0	2	0.87
End-stage CKD AKI+	0	0	
End-stage CKD AKI−	0	1	0.92
SBP AKI+	124 ± 15	137 ± 20	0.01
SBP AKI−	120 ± 13	134 ± 22	0.001
DBP AKI+	71 ± 9	79 ± 11	0.003
DBP AKI−	74 ± 12	79 ± 8	0.04
BMI AKI+	28.3 ± 4.7	27.4 ± 5.7	0.25
BMI AKI−	26.9 ± 3.9	27.9 ± 5.0	0.40
Creatinine AKI+	2.1 ± 0.7	1.7 ± 1.1	0.17
Creatinine AKI−	1.5 ± 0.4	1.6 ± 0.7	0.12
GFR AKI+	37 ± 14	46 ± 13	0.008
GFR AKI−	53 ± 15	48 ± 13	0.009
Hb AKI+	8.8 ± 1.2	13.4 ± 2.7	0.0001
Hb AKI−	9.5 ± 1.9	13.4 ± 1.8	0.0001

Data are expressed as mean ± standard deviation and absolute and relative frequencies. AKI: acute kidney injury; CVD: cardiovascular disease; CKD: chronic kidney disease; SBP: systolic blood pressure; DBP: diastolic blood pressure; BMI: body mass index; GFR: glomerular filtration rate; Hb: hemoglobin.
